# The description of cough sounds by healthcare professionals

**DOI:** 10.1186/1745-9974-2-1

**Published:** 2006-01-25

**Authors:** Jaclyn A Smith, H Louise Ashurst, Sandy Jack, Ashley A Woodcock, John E Earis

**Affiliations:** 1 North West Lung Research Centre, South Manchester Hospitals University Trust, Wythenshawe Hospital, Southmoor Rd, Manchester, M16 0DR, UK; 2 Aintree Chest Centre, University Hospital Aintree, Longmoor Lane, Liverpool, Merseyside L9 7AL, UK

## Abstract

**Background:**

Little is known of the language healthcare professionals use to describe cough sounds. We aimed to examine how they describe cough sounds and to assess whether these descriptions suggested they appreciate the basic sound qualities (as assessed by acoustic analysis) and the underlying diagnosis of the patient coughing.

**Methods:**

53 health professionals from two large respiratory tertiary referral centres were recruited; 22 doctors and 31 staff from professions allied to medicine. Participants listened to 9 sequences of spontaneous cough sounds from common respiratory diseases. For each cough they selected patient gender, the most appropriate descriptors and a diagnosis. Cluster analysis was performed to assess which cough sounds attracted similar descriptions.

**Results:**

Gender was correctly identified in 93% of cases. The presence or absence of mucus was correct in 76.1% and wheeze in 39.3% of cases. However, identifying clinical diagnosis from cough was poor at 34.0%. Cluster analysis showed coughs with the same acoustics properties rather than the same diagnoses attracted the same descriptions.

**Conclusion:**

These results suggest that healthcare professionals can recognise some of the qualities of cough sounds but are poor at making diagnoses from them. It remains to be seen whether in the future cough sound acoustics will provide useful clinical information and whether their study will lead to the development of useful new outcome measures in cough monitoring.

## Background

Cough is the commonest symptom for which patients seek medical advice [1] but the quality of cough sounds is currently largely ignored in the clinical examination of adults. Like many physical symptoms and signs in clinical medicine the value of assessing the cough sound is unclear. The inter-observer repeatability of the presence or absence of a range of respiratory physical signs falls midway between chance and total agreement [2]. However, medical textbooks describe different types of cough (i.e. dry, moist, productive, brassy, hoarse, wheezy, barking etc), implying these terms are of some clinical value. Paediatricians not uncommonly use the diagnostic value of different types of cough [3,4]. For example, whooping cough, bronchiolitis, croup, and cough associated with tracheo-oesophageal fistula have well recognised specific features. Though it is not uncommon to ask an adult patient to describe their cough during clinical assessment, one study has suggested that the patient's own description of the character, quality and timing is of no help in ascertaining the cause [5].

Acoustic analysis can be used to assess objectively the sound properties of respiratory sounds. Studies examining the waveforms of voluntary cough sounds, 'tussiphonograms', suggest they may be of diagnostic use, but extensive validation has not been performed [6]. Investigation of the acoustic properties of spontaneous cough sounds has demonstrated some significant differences between cough in different diseases [7]. Examination of the waveforms and spectrograms (frequency content) can identify features of cough sounds associated with mucus in the airways [8,9]. and wheezing sounds [7,10]. The ability of health professionals to appreciate these basic features is unknown. If such qualitative differences can be reliably recognised by the trained ear, cough quality could contribute to the clinical examination.

Currently, little is known about how those who work in adult respiratory medicine use the many descriptions of cough available. In this study we have used spontaneous cough sounds from overnight cough recordings in patients with common respiratory conditions. We have investigated how physicians and other health care professionals choose to describe cough sounds, whether they appreciate the basic sound qualities of coughs and whether they can identify diagnosis from cough. We hypothesised that the use of cough descriptors would demonstrate an ability to detect the basic sound qualities of cough but that they would be poor at patient diagnosis.

## Methods

### Study subjects

53 observers (22 respiratory physicians and 31 other health professionals) were recruited at two hospital sites (North West Lung Centre, Manchester, UK and Aintree Chest Centre, Liverpool, UK). The physicians consisted of consultants (10) and respiratory trainee registrars (12). Healthcare professionals included clinical physiologists (12), physiotherapists (11) and specialist respiratory nurses (8).

### Study design

Nine short sequences of spontaneous cough sounds (mean length 6.7 seconds) were selected from digital sound recordings and stored on a laptop computer attached to a stereo speaker system. Each sequence of cough sounds was played 3 times in succession, to groups of observers, using the same sound system. The observers completed a questionnaire for each cough sequence, identical instructions for questionnaire completion being given.

### Cough sounds

The cough sounds were selected randomly from an extensive database of spontaneous cough sounds, recorded overnight, in patients with pulmonary diseases. The quality of these coughs sounds was assessed by experienced cough research workers by listening to the cough sounds and then confirmed by sound analysis (examination of the waveforms and spectrograms). The patients' diagnosis and clinical information was not available to the experts when doing this. They were categorised as (A) cough alone (B) cough with mucus, (C) cough with wheeze, or (D) cough with wheeze and mucus (Table 1). Recordings had been made using a free field lapel microphone (AOI, ECM-1025 electret, condenser microphone) and digital recording device (Creative Labs Ltd, Singapore) at sampling rate of 16 kHz (16-bit). Recordings were made from patients with chronic obstructive pulmonary disease (COPD), asthma, idiopathic pulmonary fibrosis (IPF), laryngitis, and bronchiectasis. The diagnoses had been established by respiratory physicians in a tertiary referral centre from investigations including pulmonary functions tests, histamine challenge, and thoracic CT scans. The sound files used for this study are available as additional files 1, 2, 3, 4, 5, 6, 7, 8 and 9 (converted to mp3 format) which can be downloaded and listened to using a media player such as Windows Media Player (Microsoft Corporation).

**Table 1:** Characteristics of cough sounds; see additional files 1-9 for the sound files used in this study (converted to mp3 format).

No.	Gender	Cough with mucus	Cough with wheeze	Diagnosis	Category
1	Female	no	no	Laryngitis	A
2	Male	yes	yes	COPD/Bronchiectasis	D
3	Female	no	yes	COPD	C
4	Male	no	no	IPF	A
5	Female	no	no	IPF	A
6	Female	no	yes	Asthma	C
7	Male	no	yes	Asthma	C
8	Male	yes	no	Bronchiectasis	B
9	Male	yes	yes	COPD	D

### Sound analysis

Cough sounds were analysed using custom written software with a visual and audio output, (programmed in Matlab 6.0 Release 12, The Mathworks Inc, MA, US). Typical cough sounds contain two or three phases[6,9,10]. These phases are most commonly referred to as the first cough sound, intermediate phase and second cough sound (when present). Cough waveforms were rectified and smoothed to produce a signal envelope from which the length of the cough phases can be determined, as described elsewhere [11].

Spectral analysis was performed using the fast Fourier transform (FFT). Wheezes were defined according to CORSA guidelines (Computerized Respiratory Sound Analysis) i.e. a continuous sound, with musical characteristics, periodic waveforms, a dominant frequency >100 Hz and with a duration of >100 ms [12]. The acoustic differences between coughs with and without mucus have only previously been described from study of voluntary cough sounds [8,9]: specifically coughs with mucus have significantly longer second phases and clear vertical lines can be seen in the sound spectrum.

### Questionnaire Design and Analysis

For each cough sequence subjects were asked to identify the patient's gender, select appropriate descriptors and a diagnosis. Widely used and respected respiratory textbooks were used to collect descriptors of cough sounds [13-19]. The 10 most common descriptors were included in the questionnaire in random order (dry, moist, productive, brassy, bovine, barking, rattling, hoarse, wheezy and loose). Subjects were asked to circle the descriptors that fitted each cough sound; the selection of more than one descriptor was permitted. The opportunity was also given to make suggestions for other appropriate descriptors. Subjects were then asked to choose the most likely diagnosis from a list of 8 possibilities (asthma, COPD, bronchiectasis, idiopathic pulmonary fibrosis, vocal cord paralysis, acute laryngitis, cystic fibrosis, and tracheomalacia).

The proportions of correct observations of the gender and diagnoses were calculated. The scores for the different occupational groups were compared using a one-way ANOVA. Scores were also compared to those expected by chance alone (one sample t-test). The use of cough sound descriptors was examined in two different ways.

Firstly, the cough descriptors were grouped into those traditionally implying cough with mucus (moist, productive, rattling and loose), cough without mucus (dry, barking, hoarse) and cough with wheeze (wheezy). The choice of cough descriptors could then be compared to the acoustic analysis of the cough sounds (Tables 1 and 2.) and the proportion of responses correctly identifying the presence or absence of mucus and wheeze recorded. If the descriptors chosen were contradictory e.g. dry and rattling, the response was considered incorrect. The percentage of correct responses was then compared for different occupational groups (ANOVA).

**Table 2:** Frequency of use of cough descriptors for each cough sound (maximum score of 53 for each cough for each descriptor, if chosen by all subjects).

No.	Dry	Brassy	Rattling	Loose	Productive	Moist	Bovine	Hoarse	Wheezy	Barking
1	50	4	1	0	0	3	0	12	11	5
2	1	2	23	12	31	21	3	6	17	6
3	29	7	9	5	1	10	0	9	24	2
4	41	10	2	1	0	2	3	6	4	6
5	45	3	1	1	0	2	0	12	17	6
6	22	6	4	1	3	1	8	18	26	30
7	26	5	3	8	10	5	7	5	13	11
8	0	0	23	37	47	30	0	2	8	0
9	8	5	23	19	22	11	4	12	27	5
Totals	222	42	89	84	114	85	25	82	147	71

Secondly, the use of descriptors was further explored using cluster analysis (agglomerative hierarchical clustering) to find which cough sounds provoked the same descriptions[20]. Squared Euclidean distance was used as the measure of dissimilarity. The results are presented in the form of a dendrogram beginning with 9 clusters (one for each separate cough sound). The clustering procedure progressively groups coughs sounds by descriptors until eventually one cluster, containing all the sounds is formed. The more similar the cough sounds are (in terms of description) the more rapidly they cluster together. All statistical analyses were performed using SPSS 11.0 (Chicago) and Prism 4 (Graphpad Ltd).

## Results

### Sound analysis

**Figure 1:**
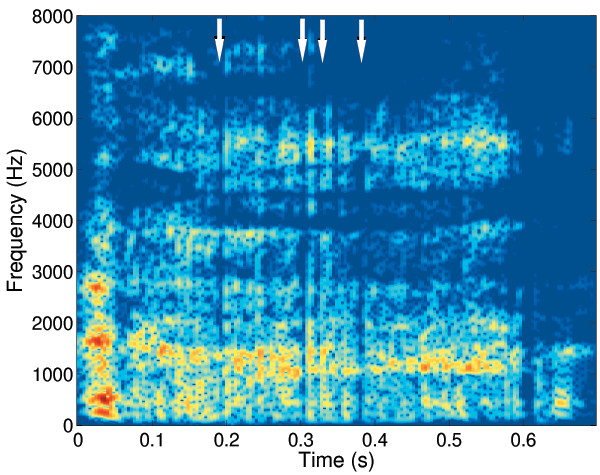
Spectrogram showing the change in frequency content over time in a male bronchiectasis cough (cough 8, no wheeze with mucus). Arrows show interruptions in sound spectrum.

**Figure 2:**
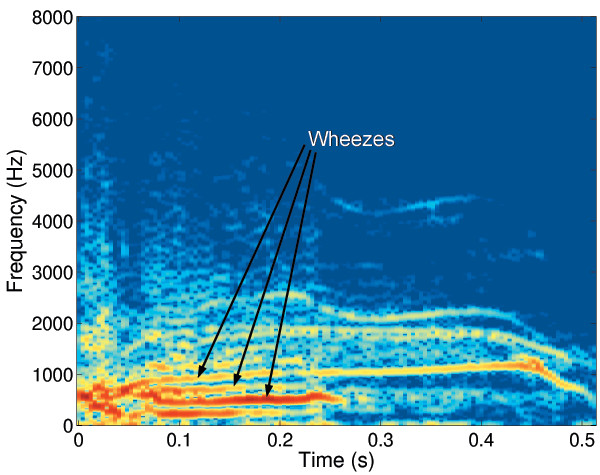
Spectrogram showing the change in frequency content over time in a female asthmatic cough (cough 6, wheeze with no mucus). Darker frequencies have higher amplitudes. Wheezing can be clearly seen represented by a series of horizontal bands.

Table 1 shows a summary of the acoustics properties of the cough sounds and the consequent categories. Analysis of the cough phases found 8 of the 9 cough sounds had a 3 phases present. The coughs with mucus had significantly longer second phase (p = 0.02) and total length (p = 0.02) in keeping with previous reports [8,9]. The spectrograms in coughs with mucus all showed clear vertical lines in the second phase as reported by Murata (Figure 1) [8], unlike those without mucus. Four coughs contained wheezes in the intermediate phase with dominant frequencies 632, 766, 1162 and 1193 Hz and durations of 1951, 756, 275 and 202 ms respectively. Figure 2 shows a typical spectrogram of wheezes within the second phase of the cough sound.

### Questionnaire responses

**Figure 3:**
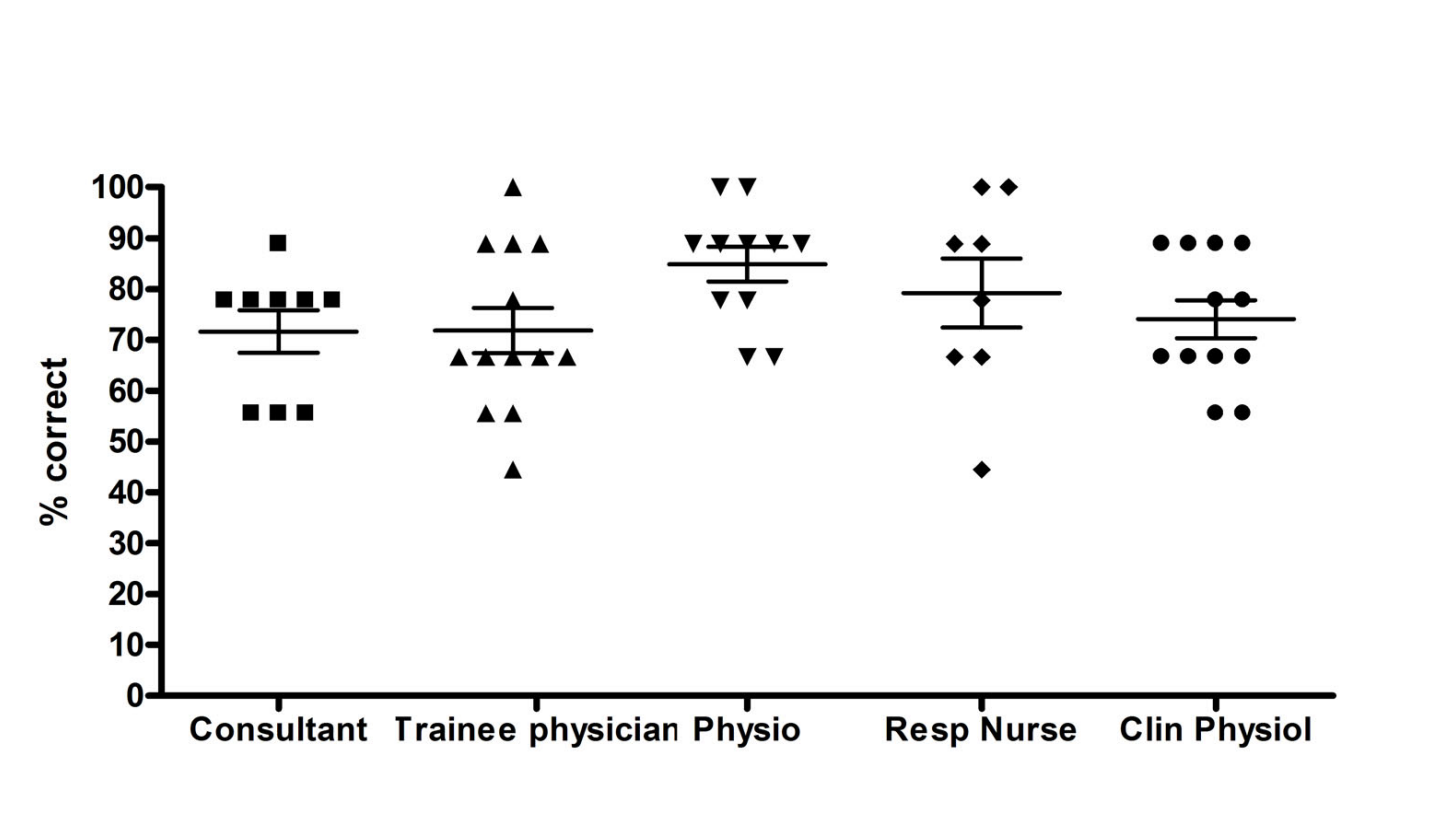
Percentage of coughs with mucus correctly identified by job title (mean with 95% confidence intervals).

**Figure 4:**
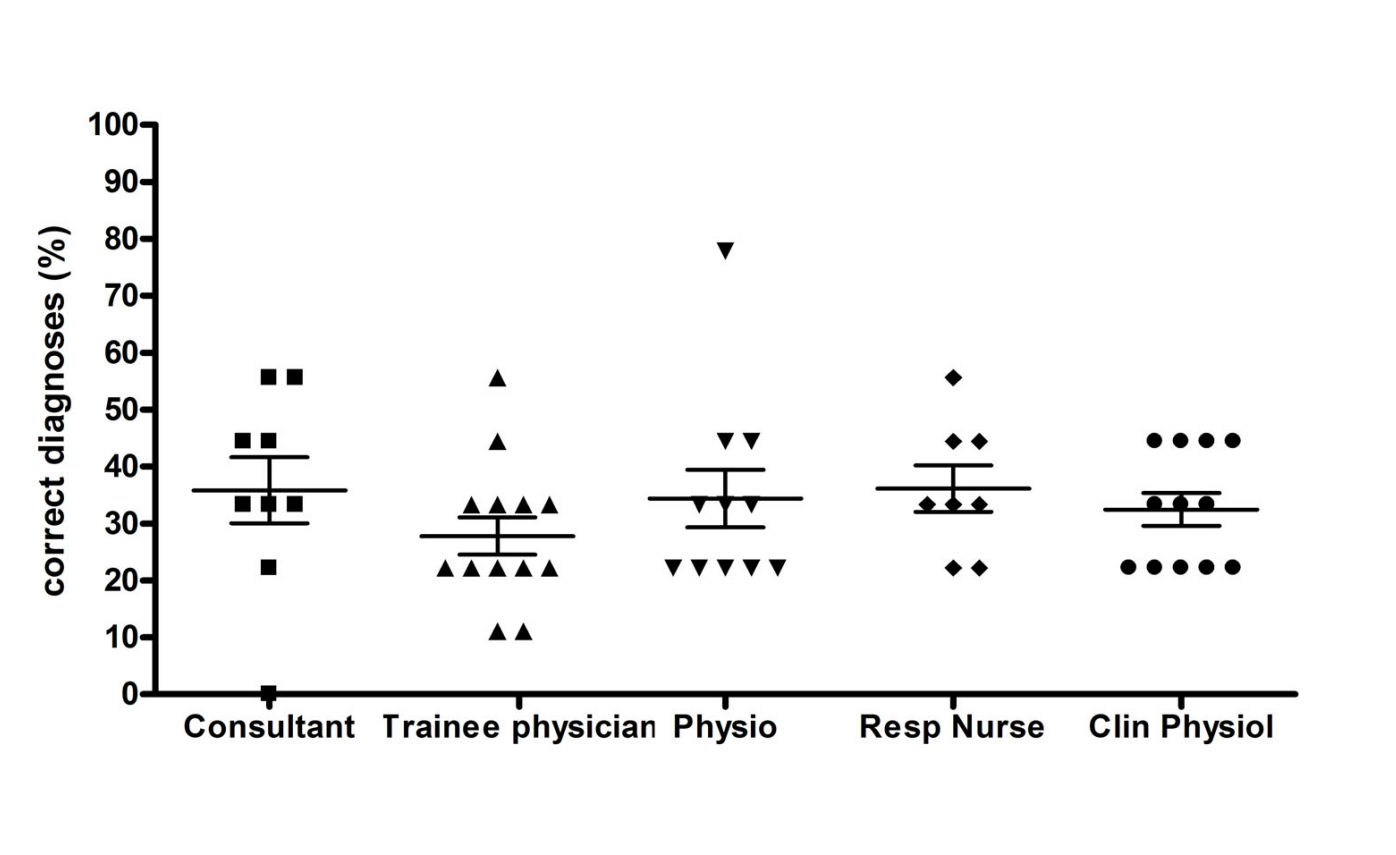
Percentage of diagnoses correct by job title (mean with 95% confident intervals).

Subjects were very good at identifying gender: a mean of 93.0% were correct, averaged across all questions (standard deviation ± 7.6%). They were also good at correctly differentiating cough with or without mucus (76.1% ± 14.8) (Figure 3) but not cough with wheeze (39.3% ± 15.0), but the ability to detect these qualities was more variable. Subjects were rarely able to use audible cough characteristics to correctly identify the clinical diagnosis from the seven diagnoses on offer (34.0% ± 29.0%), (Figure 4). Performance was still significantly better than the expected percentage correct by chance for all questions (p =< 0.01, single sample t-tests).

There were no statistically significance differences between the different occupational groups' ability to characterise basic cough quality (wheeze p = 0.54 and mucus p = 0.38) or to assign a diagnosis (p = 0.36). There was no significant correlation between the ability to recognise gender and diagnosis (r = 0.09, p = 0.54).

### Cluster analysis

The frequency of use of the cough descriptors is shown in Table 2. Dry, productive and wheezy were the most popular descriptors but a range of different descriptors were chosen for each cough sound. Eighteen other descriptors were suggested by subjects, the most common being 'irritating', 'tight', and 'hard'. These were only used on 4 occasions each; the questionnaire descriptors were used on between 42 and 222 occasions each.

**Figure 5:**
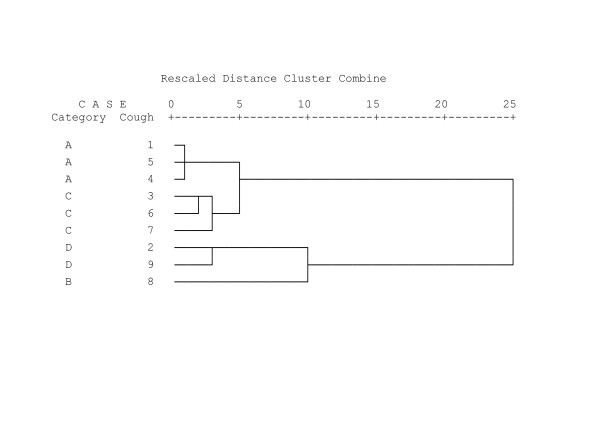
Dendrogram resulting from cluster analysis of 9 cough sequences. The cough characteristics are shown in table 1. Coughs attracting similar descriptors combine at shorter distances.

Cluster analysis was performed in order to classify cough sounds sharing similar descriptors. The results are presented in the form of a dendrogram beginning with 9 clusters (one for each separate cough sound) (Figure 5). It can be seen from the dendrogram that cough sounds 1, 4, and 5 quickly form a cluster. This group of cough sounds share the same features by acoustic analysis i.e. cough without mucus or wheeze (category A, table 1). Coughs 6, 3 and 7 (cough with wheeze and no mucus, category C) and coughs 2 and 9 (cough with mucus and wheeze, category D) cluster next and are also in the same acoustic categories. At level 10 the cough sounds form 2 distinct clusters corresponding to the division between the cough with and without mucus. Hence the cough descriptor choices cause the cough sounds to cluster by acoustic category rather than by diagnostic category.

## Discussion

This is the first study to relate the descriptions of adult cough sounds to their acoustic analysis. We have shown that health professionals are good at identifying coughs with and without mucus but are less successful at identifying wheezes in cough sounds. As predicted the ability to select the correct diagnosis for a cough from the sound alone was poor. A wide range of cough descriptors was used by our subjects and cluster analysis suggested they reflect the acoustic properties of the cough sounds rather than the diagnostic category.

Only one previous study has investigated the quality of cough sounds[21]. This study was performed in children undergoing bronchoscopy and examined the agreement between descriptions of the cough as wet or dry (by clinicians and parents) and the bronchoscopic appearances. A novel system for categorising the airway appearances was devised and good agreement was found for both clinicians and parents rating of coughs. These findings are in keeping with our study suggesting that wet or dry coughs can generally be distinguished.

The identification of wheezes in cough sounds was generally poor but the variability in performance was large with some individuals performing very well and others very badly. This may be explained by the fact that health professionals are much more accustomed to identifying wheezes superimposed on breath sounds rather than cough sounds. Subjects were able to predict accurately the gender of the patient from the cough sound; this was probably due to the differences in frequency content [22]. Subjects could have used gender to predict likely diagnosis but there was no evidence of this; there was no correlation between gender scores and diagnosis scores.

The acoustic features of wheezes are well described from the study of breath sounds and wheezes can be easily identified in the spectrogram (i.e. from the frequency components) (Figure 2). However there has been less interested in acoustic analysis of cough sounds. Only one study has described the effect of mucus on voluntary cough sounds in subjects with COPD [9]. It is our experience that these features can also be easily identified in the spectrogram of spontaneous cough sounds (Figure 1). We have not found the audiograms to be useful in identifying wheeze or mucus in cough sounds.

We included health professionals allied to medicine in our study as well as doctors because, to our knowledge, none of these groups receives any specific training in recognising the qualities of cough sounds. All participants included were working with adult respiratory patients on a daily basis and had extensive clinical experience with patients who cough. We found no significant differences in the performance of medically qualified health professionals and those qualified in professions allied to medicine. Indeed in the study by Chang[21] parents performed almost as well as clinicians in detecting cough with mucus.

It is possible that with training skills in recognising cough qualities could be improved. In a small study 5 physicians who had brief training to appreciate the features of cough waveforms from an audio-visual display could differentiate between voluntary coughs from patients with asthma and chronic bronchitis[23]. Their ability to differentiate the two conditions prior to training was not assessed and may represent the same ability to differentiate between coughs with mucus (chronic bronchitis) from coughs without mucus (asthma), demonstrated by our un-trained subjects.

This study showed that health professionals tend to use a wide range of descriptors to describe cough sounds. Many more cough descriptors were used by our participants than were found in the textbooks. A total of eighteen additional cough descriptors were suggested but none was as frequently used as the textbook terms, suggesting that these were more broadly acceptable. A hierarchical cluster analysis was used to classify cough sounds in terms of the descriptors they attracted. This type of analysis has been used in an analogous study examining the language patients use to describe breathlessness[24]. Cluster analysis of the cough descriptors produced identical categories of cough sounds to acoustic analysis. This suggests that taken together the patterns of descriptors chosen reflect an appreciation of the underlying qualities of the cough sounds rather than the underlying patient diagnosis.

That diagnosis from cough sound alone is poor is not surprising. Previous work examining voluntary cough sounds has suggested that some differences occur between diagnostic groups [6]. In our experience of acoustic analysis of spontaneous cough sounds [11] the variability of acoustic parameters between individuals is considerable and greater than that between disease groups. One of the possible explanations for this variability is that the presence of mucus in the airways during coughing or wheeze due to bronchospasm is likely to vary at different times of day, in different environments and with disease exacerbations. Therefore even if the health professional could accurately describe a cough sound during clinical assessment, this may not be of much clinical utility. Perhaps a more useful measure would be the cough quality over longer periods of time e.g. the proportion of coughs with mucus in 24 hours. It will only be possible to assess these kinds of endpoints once accurate automated cough detection systems are devised and after more extensive validation of cough sound acoustics.

## Conclusion

We conclude that health professionals are able to differentiate coughs with mucus from those without mucus, but are poor at identifying wheeze and diagnosis. The wide range of cough descriptors in use seems to be unjustified as they merely represent the basic sound qualities. This study underscores the lack of knowledge about one of the commonest symptoms in respiratory disease, the need for new techniques to measure and monitor cough, and to determine whether objective cough sound characteristics are useful.

## Declaration of competing Interests

The author(s) declare that they have no competing interests.

## Authors' contributions

JE had the original idea for the study. JE, JS, SJ and LA designed the protocol. SJ and LA collected the data from Aintree and with JS at the NWLC. JS analysed the data. All authors participated in critical discussion of the data and analyses. JS wrote the manuscript, JE and AW revised the manuscript. All authors read and approved the final manuscript.

## Supplementary Material

Additional File 1 The cough sounds used in this study have been provided in mp3 format and can be downloaded and listened to using a media player such as Windows Media Player (Microsoft). Cough1.mp3. Cough2.mp3. Cough3.mp3. Cough4.mp3. Cough5.mp3. Cough6.mp3. Cough7.mp3. Cough8.mp3. Cough9.mp3. Click here for file

Additional file 2 The cough sounds used in this study have been provided in mp3 format and can be downloaded and listened to using a media player such as Windows Media Player (Microsoft). Cough1.mp3. Cough2.mp3. Cough3.mp3. Cough4.mp3. Cough5.mp3. Cough6.mp3. Cough7.mp3. Cough8.mp3. Cough9.mp3. Click here for file

Additional file 3 The cough sounds used in this study have been provided in mp3 format and can be downloaded and listened to using a media player such as Windows Media Player (Microsoft). Cough1.mp3. Cough2.mp3. Cough3.mp3. Cough4.mp3. Cough5.mp3. Cough6.mp3. Cough7.mp3. Cough8.mp3. Cough9.mp3. Click here for file

Additional file 4 The cough sounds used in this study have been provided in mp3 format and can be downloaded and listened to using a media player such as Windows Media Player (Microsoft). Cough1.mp3. Cough2.mp3. Cough3.mp3. Cough4.mp3. Cough5.mp3. Cough6.mp3. Cough7.mp3. Cough8.mp3. Cough9.mp3. Click here for file

Additional file 5 The cough sounds used in this study have been provided in mp3 format and can be downloaded and listened to using a media player such as Windows Media Player (Microsoft). Cough1.mp3. Cough2.mp3. Cough3.mp3. Cough4.mp3. Cough5.mp3. Cough6.mp3. Cough7.mp3. Cough8.mp3. Cough9.mp3. Click here for file

Additional file 6 The cough sounds used in this study have been provided in mp3 format and can be downloaded and listened to using a media player such as Windows Media Player (Microsoft). Cough1.mp3. Cough2.mp3. Cough3.mp3. Cough4.mp3. Cough5.mp3. Cough6.mp3. Cough7.mp3. Cough8.mp3. Cough9.mp3. Click here for file

Additional file 7 The cough sounds used in this study have been provided in mp3 format and can be downloaded and listened to using a media player such as Windows Media Player (Microsoft). Cough1.mp3. Cough2.mp3. Cough3.mp3. Cough4.mp3. Cough5.mp3. Cough6.mp3. Cough7.mp3. Cough8.mp3. Cough9.mp3. Click here for file

Additional file 8 The cough sounds used in this study have been provided in mp3 format and can be downloaded and listened to using a media player such as Windows Media Player (Microsoft). Cough1.mp3. Cough2.mp3. Cough3.mp3. Cough4.mp3. Cough5.mp3. Cough6.mp3. Cough7.mp3. Cough8.mp3. Cough9.mp3. Click here for file

Additional file 9 The cough sounds used in this study have been provided in mp3 format and can be downloaded and listened to using a media player such as Windows Media Player (Microsoft). Cough1.mp3. Cough2.mp3. Cough3.mp3. Cough4.mp3. Cough5.mp3. Cough6.mp3. Cough7.mp3. Cough8.mp3. Cough9.mp3. Click here for file
